# Estimating Geographical Variation in the Risk of Zoonotic *Plasmodium knowlesi* Infection in Countries Eliminating Malaria

**DOI:** 10.1371/journal.pntd.0004915

**Published:** 2016-08-05

**Authors:** Freya M. Shearer, Zhi Huang, Daniel J. Weiss, Antoinette Wiebe, Harry S. Gibson, Katherine E. Battle, David M. Pigott, Oliver J. Brady, Chaturong Putaporntip, Somchai Jongwutiwes, Yee Ling Lau, Magnus Manske, Roberto Amato, Iqbal R. F. Elyazar, Indra Vythilingam, Samir Bhatt, Peter W. Gething, Balbir Singh, Nick Golding, Simon I. Hay, Catherine L. Moyes

**Affiliations:** 1 Spatial Ecology & Epidemiology Group, Oxford Big Data Institute, Li Ka Shing Centre for Health Information and Discovery, University of Oxford, Oxford, United Kingdom; 2 Malaria Atlas Project, Oxford Big Data Institute, Li Ka Shing Centre for Health Information and Discovery, University of Oxford, Oxford, United Kingdom; 3 Institute of Health Metrics and Evaluation, University of Washington, Seattle, Washington, United States of America; 4 Molecular Biology of Malaria and Opportunistic Parasites Research Unit, Department of Parasitology, Faculty of Medicine, Chulalongkorn University, Bangkok, Thailand; 5 Department of Parasitology, Faculty of Medicine, University of Malaya, Kuala Lumpur, Malaysia; 6 Wellcome Trust Sanger Institute, Hinxton, United Kingdom; 7 Medical Research Council (MRC) Centre for Genomics and Global Health, University of Oxford, Oxford, United Kingdom; 8 Wellcome Trust Centre for Human Genetics, University of Oxford, Oxford, United Kingdom; 9 Eijkman-Oxford Clinical Research Unit, Jakarta, Indonesia; 10 Department of Infectious Disease Epidemiology, Imperial College London, London, United Kingdom; 11 Malaria Research Centre, Universiti Malaysia Sarawak, Kuching, Sarawak, Malaysia; 12 Department of BioSciences, University of Melbourne, Parkville, Victoria, Australia; Imperial College London, UNITED KINGDOM

## Abstract

**Background:**

Infection by the simian malaria parasite, *Plasmodium knowlesi*, can lead to severe and fatal disease in humans, and is the most common cause of malaria in parts of Malaysia. Despite being a serious public health concern, the geographical distribution of *P*. *knowlesi* malaria risk is poorly understood because the parasite is often misidentified as one of the human malarias. Human cases have been confirmed in at least nine Southeast Asian countries, many of which are making progress towards eliminating the human malarias. Understanding the geographical distribution of *P*. *knowlesi* is important for identifying areas where malaria transmission will continue after the human malarias have been eliminated.

**Methodology/Principal Findings:**

A total of 439 records of *P*. *knowlesi* infections in humans, macaque reservoir and vector species were collated. To predict spatial variation in disease risk, a model was fitted using records from countries where the infection data coverage is high. Predictions were then made throughout Southeast Asia, including regions where infection data are sparse. The resulting map predicts areas of high risk for *P*. *knowlesi* infection in a number of countries that are forecast to be malaria-free by 2025 (Malaysia, Cambodia, Thailand and Vietnam) as well as countries projected to be eliminating malaria (Myanmar, Laos, Indonesia and the Philippines).

**Conclusions/Significance:**

We have produced the first map of *P*. *knowlesi* malaria risk, at a fine-scale resolution, to identify priority areas for surveillance based on regions with sparse data and high estimated risk. Our map provides an initial evidence base to better understand the spatial distribution of this disease and its potential wider contribution to malaria incidence. Considering malaria elimination goals, areas for prioritised surveillance are identified.

## Author Summary

*Plasmodium knowlesi* is a malaria parasite found in wild monkey populations and transmitted from this animal reservoir to humans *via* infected mosquitoes. It causes severe and fatal disease in humans, and is the most common cause of malaria in parts of Malaysia. The geographical distribution of this disease is largely unknown because it is often misdiagnosed as one of the human malarias. Human malaria parasites are primarily transmitted between humans *via* mosquitoes and are not frequently transmitted from other animals to humans. Many countries in Southeast Asia, where *P*. *knowlesi* infections have been reported, are making progress towards eliminating the human malarias. Understanding the geographical distribution of *P*. *knowlesi* is important for identifying areas where malaria transmission will continue after the human malarias have been eliminated. In locations that have high volumes of *P*. *knowlesi* infection data, we modelled patterns of variation in the data linked to environmental predictors, and used this to estimate *P*. *knowlesi* infection risk in locations where data is lacking. The resulting map represents an initial evidence-base for identifying areas of human disease risk that should be prioritized for surveillance, particularly in the context of malaria elimination in the region.

## Introduction

Malaria cases caused by the simian parasite, *Plasmodium knowlesi*, have been identified in at least nine Southeast Asian countries. In many parts of Malaysia, this parasite is the most common cause of malaria [1] and can lead to severe and fatal disease [2–4]. Despite the potential severity of infection [1, 4–8], diagnostics that identify *P*. *knowlesi* are not routinely used. Unless blood samples are tested using expensive nested PCR-based diagnostics, cases of *P*. *knowlesi* are often misdiagnosed by microscopy as one of the human malarias, principally *P*. *malariae* or *P*. *falciparum* [9–11].

*Plasmodium knowlesi* infection is routinely considered as a potential causal pathogen of malaria cases in three countries: Malaysia, Brunei and Singapore (hereafter a region referred to as MBS), the latter two having already eliminated the human malarias. *Plasmodium knowlesi* malaria cases have also been reported in Cambodia, Indonesia, Myanmar, the Philippines, Thailand and Vietnam [8, 12, 13], but sampling has been limited and the full geographical extent of disease risk across most of the region, including within these countries, is unknown.

Understanding the geographical distribution of *P*. *knowlesi* is important to identify areas where residual malaria transmission could remain once the human malarias, namely *P*. *falciparum*, *P*. *vivax*, *P*. *malariae* and *P*. *ovale*, have been eliminated [14]. Human malaria parasites are primarily transmitted between humans *via* mosquitoes and are not frequently transmitted from other animals to humans. Many countries in Southeast Asia, including Malaysia, the Philippines, Thailand and Vietnam, are currently in the process of eliminating the human malarias [15]. Current control measures that reduce these malarias include mass anti-malarial drug administration, the provision of insecticide-impregnated bed nets (ITNs) and indoor residual spraying of houses (IRS). These control measures do not, however, target transmission of the parasite within populations of the reservoir host species so *P*. *knowlesi* populations will not be eliminated. Further, ITNs and IRS are unlikely to offer the same degree of personal protection to humans, or community protection through reductions in mosquito longevity, since the vectors for *P*. *knowlesi* bite and rest outdoors [16]. If the presence of *P*. *knowlesi* is not considered when elimination strategies are developed, the impact of elimination measures and reduction in overall malaria cases in these areas will not match projections.

In this study, we produced the first map of the geographical distribution of *P*. *knowlesi* malaria, using a niche modelling approach previously applied to the mapping of other vector-borne and zoonotic diseases, including dengue [17], the Leishmaniases [18], Ebola virus disease [19], Lassa fever [20], Marburg virus disease [21], Crimean-Congo hemorrhagic fever [22], and Zika virus [23]. Niche models are able to combine information on locations where diseases have been recorded with geographic data on environmental and socioeconomic factors hypothesized to affect disease transmission [24]. Once the model has been fitted, the potential presence of the disease can be predicted in regions where it has yet to be reported.

To identify regions at risk from a disease with reservoirs in multiple host species, transmitted by multiple vector species, this modelling approach needs to be further refined. The spatial distribution of such diseases is restricted to locations where all species required for transmission coincide, so it is important to consider the distributions of these species [25]. The work presented here builds on previous work that assessed the evidence for the limits of transmission [12]. Here we refine those spatial limits and investigate the variation in risk within them. We recently defined the fine-scale species distributions of the known and putative reservoirs and vectors of *P*. *knowlesi* [26], including the main macaque species identified as natural hosts of *P*. *knowlesi*, *Macaca fascicularis* and *M*. *nemestrina* [27–31], and several anopheline mosquito species, all from the Leucosphyrus Group, implicated as vectors of *P*. *knowlesi* [16, 32–35]. Our maps of these species are useful for defining the limits of zoonotic transmission, but an index of disease risk cannot be extrapolated directly from reservoir/vector maps. While co-occurrence of reservoir and vector species involved in a zoonotic disease system is necessary for transmission, it is not always sufficient, as many other factors contribute. We have developed a model that incorporates the relationships between *P*. *knowlesi* infection and the distributions of the reservoir and vector species, along with a range of other potential risk factors, to produce fine-scale evidence-based predictions of relative zoonotic *P*. *knowlesi* transmission risk.

The final output provides an initial map that aims to identify locations where disease surveillance and epidemiological investigations would be most informative to improve our understanding of disease risk.

## Methods

### Overview

**Fig 1:**
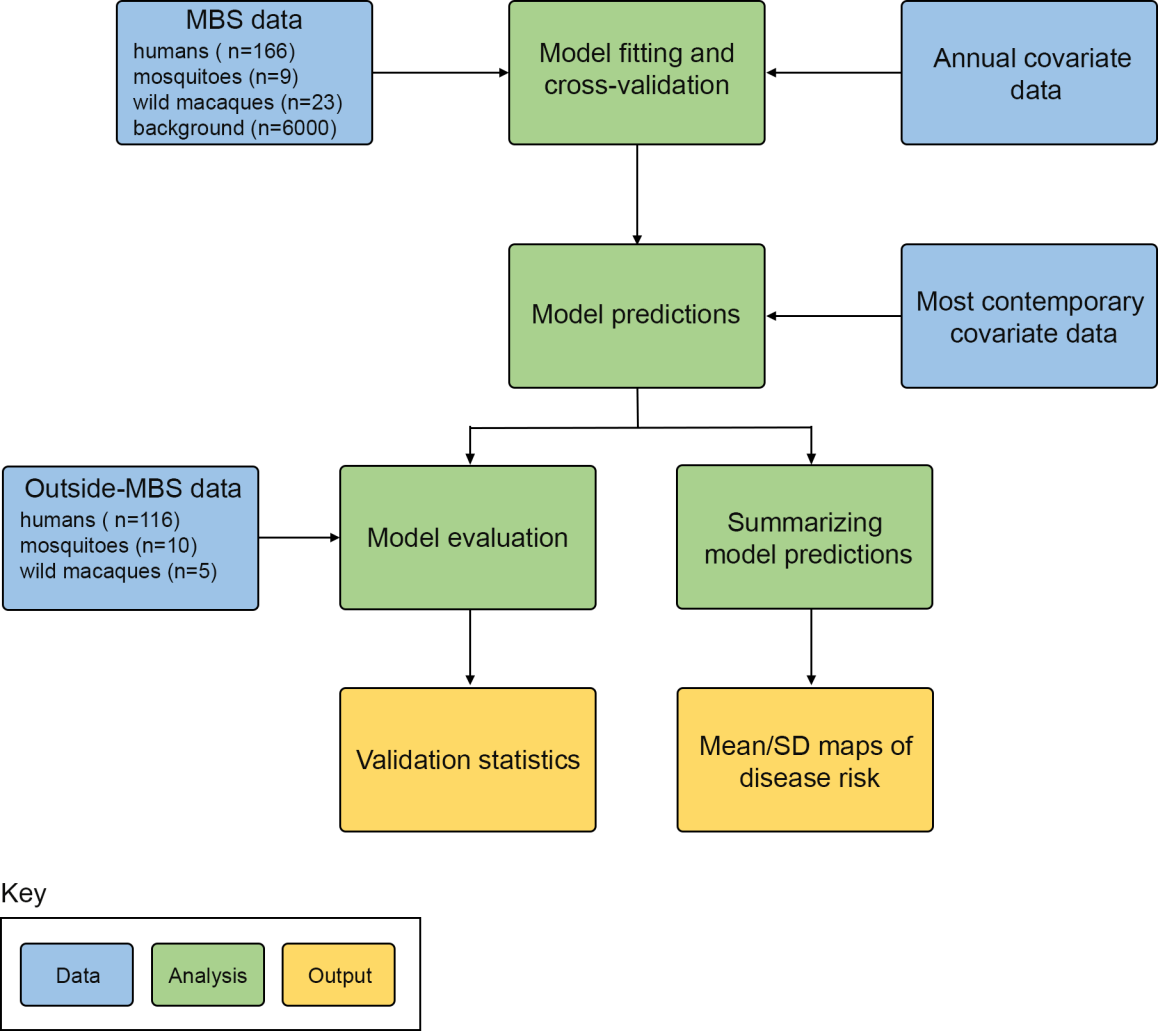
Schematic overview of the methods. Blue boxes describe input data, green boxes denote analyses, and yellow boxes represent final outputs. MBS = Malaysia, Brunei and Singapore.

A schematic of the process we followed is given in Fig 1. We collated and geo-positioned records of *P*. *knowlesi* infections in humans, and reservoir and vector species, from a variety of sources. The study area was a rectangle encompassing the locations of confirmed or putative *P*. *knowlesi* infections plus a minimum buffer zone of 300 km, giving an area from northeast Bangladesh to southwest Papua New Guinea. A map predicting the human risk of *P*. *knowlesi* malaria at every square (pixel) in a 5 km × 5 km grid was generated using an ensemble of boosted regression tree (BRT) models to carry out a niche modelling analysis. The model used the database of geo-positioned occurrence points for *P*. *knowlesi* infections combined with 19 gridded datasets of environmental and socio-economic explanatory covariates as well as probabilistic species distributions for *M*. *nemestrina*, *M*. *fascicularis* and the Leucosphyrus Group.

Datasets comprising ad-hoc reports of disease occurrence (as opposed to data from planned region-wide surveys) are subject to spatial bias in reporting rates, which if unaccounted for may result in elevated risk predictions in the areas most likely to report [36]. Reporting bias is likely to be more pronounced for *P*. *knowlesi* malaria since significant resources are required to accurately diagnose infection and *P*. *knowlesi* infection is not routinely considered a possible cause of malaria in the region, with the exception of MBS.

A model was therefore fitted using data only from MBS (the model training region), where we could account for reporting bias through our selection of background data. This model was then used to predict the human risk of *P*. *knowlesi* infection across Southeast Asia. To assess the model’s predictive capacity outside its training region, we tested its performance on a set of disease presence and absence records from locations outside MBS. We also generated a multivariate environmental similarity surface to identify regions where the model was required to extrapolate to environments not found within MBS, and therefore evaluate the appropriateness of inferring risk in those regions.

### Occurrence dataset

Records of *P*. *knowlesi* presence or absence for Southeast Asia were obtained from reports in the published literature from 2004 to 2015, which were validated through personal communications with the authors to confirm details. Each presence record contains the coordinates of a point location or an area greater than 25 km^2^ (polygons) where a human, macaque or mosquito infection was confirmed using specific diagnostics that are able to distinguish *P*. *knowlesi* from the other *Plasmodium spp*. Presence points were excluded if *P*. *knowlesi* presence in the surrounding area (within 300 km radius) was not verified by a second, independent laboratory. Each absence record contains the coordinates for a site where an appropriate diagnostic for *P*. *knowlesi* was used, but no infections were found in a sample size of at least 500 individuals, or where no malaria cases were reported across an administrative division in 2012 [37]. Further details regarding data assemblage are included in the Supporting Information along with the complete data (S1 and S2 Files).

### Explanatory covariates

Nineteen 5 km × 5 km gridded data surfaces of a range of environmental and socio-economic factors, along with predicted reservoir and vector species distributions, were used as explanatory covariates (Table 1). No prior assumptions were made about the nature of any relationships between these covariates and disease risk. Further details regarding the construction of each covariate data surface is provided in the Supporting Information along with plots of each surface (S1 File, and S1 and S2 Figs).

**Table 1:** Explanatory covariates included in the analysis and their data source.

Covariate	Data source
Open shrublands, woody savannas, savannas, grasslands, wetlands, croplands, and cropland mosaics land cover classes (proportional)	MODIS land cover product [38]
Intact forest cover (proportional)	MODIS land cover product [38] Intact Forest Landscape [39]
Disturbed forest cover (proportional)	MODIS land cover product [38] Intact Forest Landscape [39]
Elevation	Shuttle Radar Topography Mission [40]
Temperature suitability index for *Plasmodium falciparum* transmission	Gething *et al*. 2011 [41]
Tasseled cap wetness, a measure of surface moisture (mean and standard deviation)	Gap-filled MODIS satellite data [42, 43]
Tasseled cap brightness, a measure of moisture on bare surfaces (standard deviation)	Gap-filled MODIS satellite data [42, 43]
Human population density	WorldPop [44] and Gridded Population of the World [45]
Urban accessibility	European Commission Joint Research Centre [46]
Species distributions for *Macaca fascicularis*, *M*. *nemestrina* and the Leucosphyrus Group	Moyes *et al*. 2016 [26]

### Model fitting

To carry out the niche mapping analysis, we fitted an ensemble of boosted regression tree (BRT) models using the gbm R package [47]. This BRT approach has the ability to fit complex nonlinear responses including high-dimensional interactions between explanatory variables [48], has been shown to have high predictive accuracy [24] and has been previously applied to disease distribution mapping [17–23].

Boosted regression trees combine two algorithms: regression trees (which repeatedly split the data into two groups using a randomly selected predictor variable for each split) and boosting (which additively fits trees to the data, gradually prioritizing poorly modelled data to produce a set of trees that maximally reduce the loss function), to examine and quantify the relationship between explanatory variables and the response data [48]. The core setup used has been described previously [17, 19, 48]. The changes made to the method for the work presented here addressed sampling bias in the infection reports within MBS, incorporated host and vector data, allowed temporal changes in land cover to be incorporated, and improved handling of polygon data.

Rather than exclusively using synoptic (averaged across time) covariate values for each of the occurrence locations irrespective of the occurrence date, we incorporated annual data surfaces describing land cover and reservoir and vector distributions from 2001 to 2012. This was necessary to account for the substantial changes in land cover that have occurred in the region over the study period due to deforestation [49], which is hypothesized to have impacted the distribution of the reservoir and vector species of *P*. *knowlesi* [50]. Using the annual land cover data surfaces, disease occurrence data collected between 2001 and 2012 were matched with covariate values for the relevant year; most data points (76%) fell within this time period. Covariate values for 2001 were used for occurrence data prior to this date and covariate values for 2012 were used for post-2012 data. Final predictions were made to the most contemporary covariate values available to represent the current distribution of disease risk.

We used a binomial likelihood function for the BRT model in order to incorporate both presence and absence records. Whilst records of disease absence are highly informative, they are rare because they require significantly greater sampling effort to ensure their reliability compared to presence data [51], especially for diseases like *P*. *knowlesi* malaria where the appropriate diagnostics are rarely used. We therefore supplemented the dataset with a large number of background records to represent areas where the disease has not been reported within MBS. Six thousand background points were generated in total [51] with the same proportion of human, macaque and mosquito background points as the presence dataset. It has been demonstrated that predictive accuracy of presence-background niche models can be improved by selecting background data with similar spatial bias to the occurrence records [36]. Human infection background points were generated by randomly sampling across MBS, biased towards human population density, since more populous areas have a greater probability of reporting at least one case. This method was also used to generate background points for the mosquito infection data since all studies that looked for *P*. *knowlesi* infections in vector species, selected study locations based on the presence of human *P*. *knowlesi* cases in the immediate vicinity. Background points for the macaque infection data were randomly sampled from a macaque occurrence and mammal survey dataset that reflected the bias in locations chosen for macaque studies [26]. Covariate values for the specific times and locations of the background data were then extracted. Prior to covariate extraction, human and vector background points were assigned a year randomly sampled from the temporal distribution of presence points for each species.

Since the occurrence dataset included data from humans, macaques and mosquitoes, a joint model was fitted for human, macaque and mosquito hosts that enabled all available infection data to be leveraged, whilst not constraining the model to assume that the distribution of infection risk would be identical for all three host organisms. As BRTs can fit high-dimensional interactions, the joint model is able to fit different environmental responses for each host organism, or if there is no difference in the signal, to fit the same response for all of them.

To increase the robustness of model predictions and quantify model uncertainty, we fitted an ensemble of 500 BRT models (sub-models), each trained to a separate bootstrap dataset randomly sampled with replacement from the complete presence/background dataset. To incorporate uncertainty in the precise location of infection for polygon occurrence records, each bootstrap randomly selected a 5 km × 5 km pixel within each polygon. Further information on model fitting can be found in the Supporting Information (S1 File) and the R code used to carry out the analysis is freely available at (https://github.com/fshearer/pk_parasite).

### Model prediction and evaluation

To generate the final prediction map, a mean predicted value of suitability for infection was calculated across the 500 sub-models (each fitted using occurrence and covariate data from within MBS) for each 5 km × 5 km pixel within and outside MBS.

The model’s predictive performance was evaluated using the area under the receiver operator curve (AUC) statistic, *i*.*e*. the area under a plot of the true positive rate versus false positive rate, reflecting the ability to discriminate between presence and background records [52]. The overall statistic was calculated as the mean of the AUCs for each of the 500 sub-models, calculated under 10-fold cross validation.

While each sub-model in the BRT ensemble was fitted using occurrence and background data from MBS (the model training region), the goal of our analysis was to predict to a much broader study area from northeast Bangladesh to southwest Papua New Guinea. To assess the model’s predictive performance outside its training region, a separate AUC value was calculated for each sub-model using a validation dataset made up of presence and absence records from locations outside MBS. This AUC was calculated for each sub-model and then averaged across all 500 sub-models in the ensemble. Further information regarding the calculation of the AUC statistics is provided in the Supporting Information (S1 File).

### Multivariate environmental similarity surface

The geographic regions outside MBS encompasses environments beyond the ranges of covariate values sampled within the training dataset. Model predictions to such environments are inherently less reliable than interpolations made to areas with environments within the range of covariate values in the training dataset. Thus it is important to assess the environmental similarities and differences between model training and prediction regions [53].

To investigate whether predictions to new geographic regions required extrapolation to covariate values beyond the range of the model training data, we computed and plotted a multivariate environmental similarity surface (MESS) [54] using R packages “dismo” [55] and “raster” [56]. This surface represents the similarity of the environment at each location to the covariate values at the presence and background locations in MBS (the reference data). The MESS calculation produces negative values for novel environments, locations where at least one covariate has a value that is outside the range of reference values (hereafter extrapolation), and positive values for locations within this range (hereafter interpolation). We converted the raw MESS output into a binary map indicating areas in which model predictions used interpolation versus extrapolation.

### Mask

The model output was restricted to areas within the range of species known and hypothesized to be required for zoonotic transmission (*i*.*e*. the overlap in range maps of at least one reservoir and vector species), using previously reported species range maps [26].

A high resolution map for the zone of zoonotic transmission was also generated (S3 Fig) using existing species distribution maps and occurrence datasets [26]. Threshold environmental suitability values for each of the species distribution maps for *M*. *fascicularis*, *M*. *nemestrina*, *M*. *leonina*, and the Leucosphyrus Group were determined to incorporate 90% of presence points in each species’ respective occurrence database. We used these thresholds to classify each continuous species map as either present or absent for every 5 km × 5 km pixel in the study region. These maps were combined to produce a final binary output showing areas of spatial co-occurrence of all species required for zoonotic, vector-borne transmission to humans *i*.*e*. presence of at least one macaque species plus at least one member of the Leucosphyrus Group.

## Results

**Fig 2:**
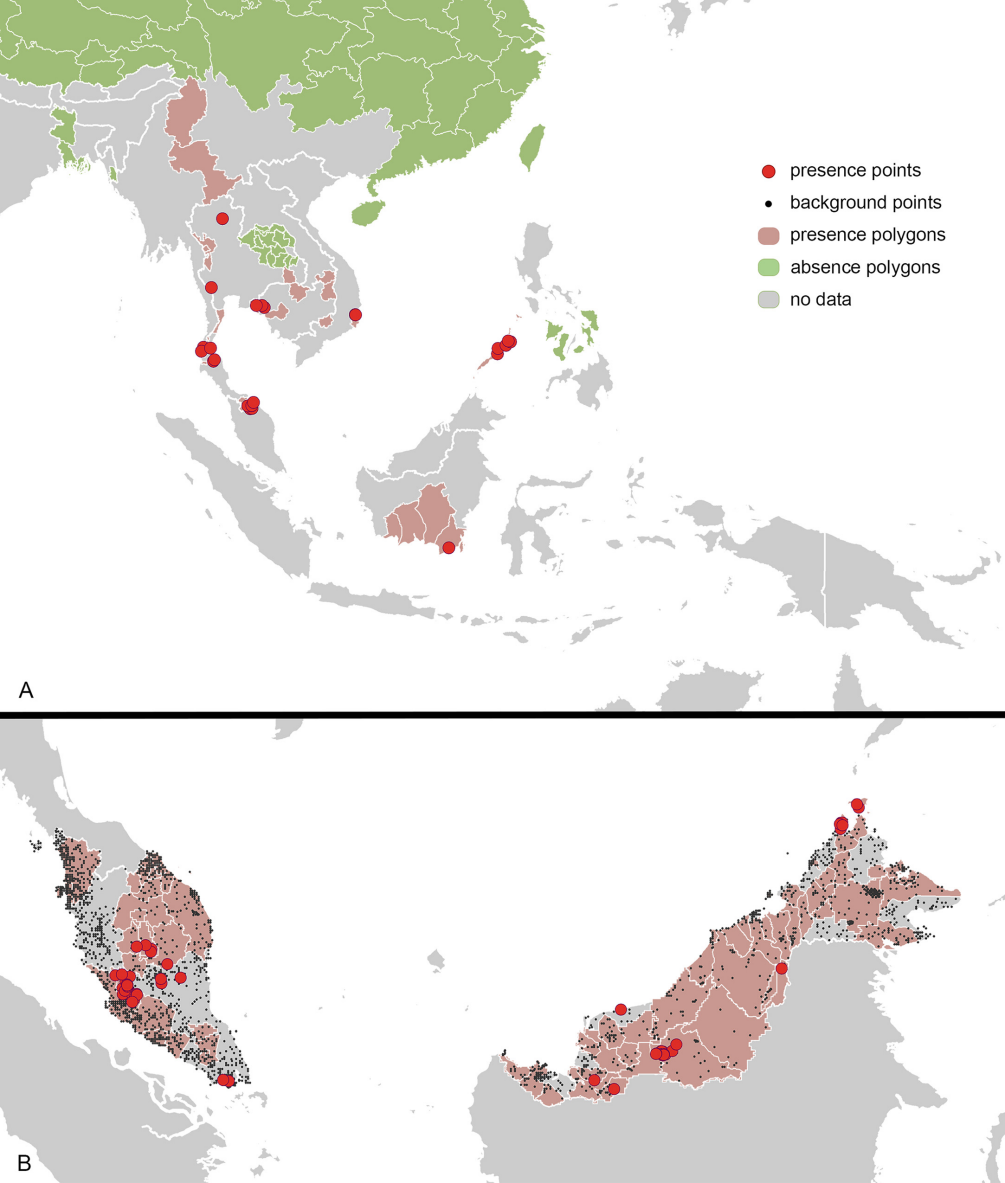
Occurrence data used for model fitting and evaluation. **A.** Location of presence and absence points/polygons outside Malaysia, Brunei and Singapore used for model evaluation. **B.** Location of presence and absence points/polygons as well as background points from Malaysia, Brunei and Singapore used for model fitting.

A total of 439 *P*. *knowlesi* occurrence records were identified, consisting of 301 presence and 138 absence records. The evaluation dataset (records falling outside MBS) totaled 131 records, comprising 29 point locations and 102 polygons (Fig 2A). The occurrence dataset used for model fitting (records falling within MBS) totaled 198 records, corresponding to 62 unique point locations and 136 polygons (Fig 2B). The model fitting dataset consisted of human (166), monkey (23) and mosquito (9) occurrence records.

**Fig 3:**
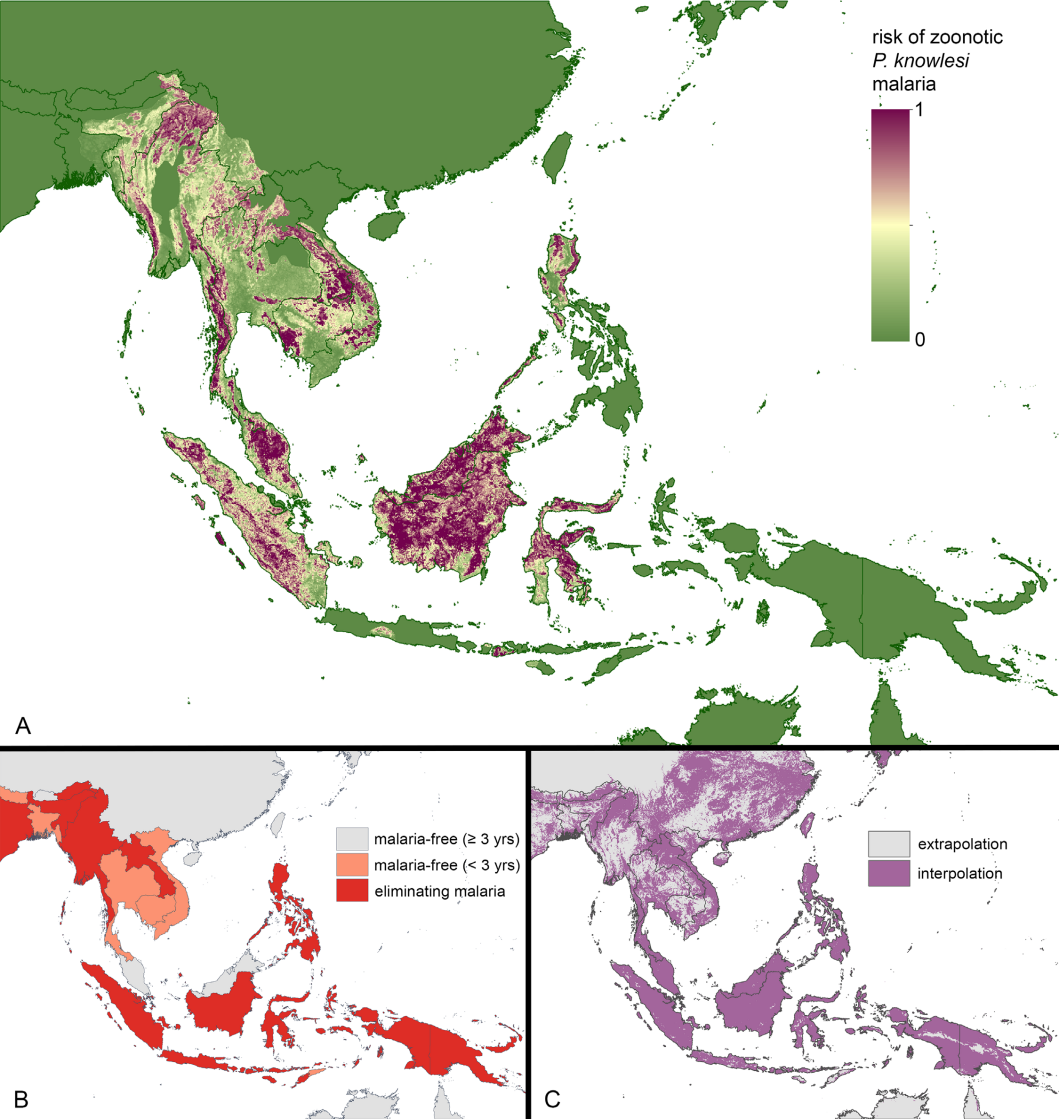
Maps of *Plasmodium knowlesi* malaria risk, human malaria elimination status, and model extrapolation versus interpolation. **A.** Predicted risk of *P*. *knowlesi* malaria ranging from low to high risk. **B.** Countries projected to be malaria-free, eliminating malaria, or controlling malaria by 2025 (Map sourced from the University of California San Francisco Global Health Group’s Malaria Elimination Initiative) **C.** Comparison of environments in Malaysia, Brunei and Singapore (the model training region) with those across the rest of Southeast Asia, using all covariates and the multivariate environmental similarity surface (MESS) methods. The map distinguishes between areas of model interpolation and areas where the model was required to extrapolate to novel environments.

The model predictions for the geographical variation in *P*. *knowlesi* infection risk in humans are displayed in Fig 3A. Overall, 10-fold cross validation statistics for the model ensemble (calculated using model training data) indicated high predictive performance with an AUC of 0.833 (SE ± 0.002). A map of model uncertainty is displayed in the Supporting Information (S4 Fig).

The model output was restricted to areas within the geographic range of the species required for zoonotic transmission. An unmasked version of the mean output, showing relative suitability for zoonotic *P*. *knowlesi* transmission, is provided in the Supporting Information (S5 Fig).

The predicted map is presented alongside a projection of malaria eliminating countries in the year 2025 (Fig 3B) and together the two maps show countries where *P*. *knowlesi* transmission may persist after the human malarias are eliminated. The elimination projections, generated by the University of California San Francisco Global Health Group’s Malaria Elimination Initiative, are based on current national and regional goals as well as recent epidemiological trends for the human malarias, principally *P*. *falciparum* and *P*. *vivax* [57].

Within MBS, our model predicted considerable spatial variation in risk of *P*. *knowlesi* infection, with areas ranging from relatively low risk to high risk predicted within Peninsular Malaysia, and both Sabah and Sarawak States of Malaysian Borneo (Fig 3A).

The model also predicts areas of high risk for *P*. *knowlesi* infection in a number of countries that are forecast to be malaria-free by 2025 (Malaysia, Cambodia, Thailand and Vietnam) as well as countries projected to be eliminating malaria (Myanmar, Laos, Indonesia and the Philippines) (Fig 3B). Large areas of high risk were predicted in Myanmar, Laos, Cambodia and Indonesia, with smaller areas predicted in Vietnam and Thailand. Human cases of *P*. *knowlesi* infection have been reported across this broad area (Fig 2A).

Regions for which we have no field data include areas of high predicted-risk, for example, eastern and western parts of Indonesia, and far eastern parts of India, although the predictions for the latter depend on whether *M*. *leonina*, included in the range of zoonotic transmission, is indeed a reservoir species (Fig 3A).

Our predictions outside MBS are a result of both interpolation within the environmental space and extrapolation. The binary MESS map (Fig 3C) shows that model extrapolation to novel environments occurred in large regions in Cambodia, Vietnam, Thailand, Myanmar, India, and the Andaman and Nicobar Islands, indicating that predictions in these areas should be interpreted with caution. The model did, however, demonstrate high predictive performance at sites outside the model-training region, with an AUC statistic of 0.796 (SE ± 0.003) calculated using 131 presence/absence locations from the evaluation dataset (Fig 2A). The predicted values for the evaluation data are presented in the Supporting Information (S6 Fig).

The main predictors for *P*. *knowlesi* infection risk were urban accessibility, human population density, elevation, proportional cover of land with croplands and environmental suitability for *M*. *nemestrina*. Marginal effect plots for each of these covariates are displayed in the Supporting Information (S7 Fig).

## Discussion

Using a niche model informed by a spatial database of *P*. *knowlesi* infections, a range of environmental and socio-economic data, and reservoir and vector species distributions, we have produced the first map of the predicted geographical distribution for *P*. *knowlesi* malaria. Empirical data on *P*. *knowlesi* presence or absence is lacking for most of Southeast Asia and this map provides an initial evidence base to prioritize areas for disease surveillance and future epidemiological investigations.

The predictive performance of the model was high and it also had a high capacity to predict suitability for infection in regions outside MBS. The latter result should, however, be treated with caution as data for model evaluation was only available from a limited number of locations outside MBS, and the selection of locations for which *P*. *knowlesi* has been tested is likely to be biased.

Another important caveat is the large area to which model predictions were made, relative to the model training region, since this required the model to extrapolate to some novel environments (see Fig 3C). Extrapolated predictions are inherently less reliable than those made in areas of interpolation and include large parts of continental Asia. Sampling for *P*. *knowlesi* infections in areas of extrapolation is likely to have the biggest impact on improving the disease risk predictions.

The final map therefore shows the risk of zoonotic *P*. *knowlesi* transmission from known reservoirs (specifically *M*. *nemestrina* and *M*. *fascicularis)* and vectors of the *Anopheles leucosphyrus* Group. If human-to-human transmission were occurring, this form of the disease is likely to have a different niche to the zoonotic disease, *i*.*e*. a different relationship with environmental, socioeconomic and biological factors. Thus our model is not appropriate to predict human-to-human transmission risk.

It is also important to note that the limits of zoonotic transmission, within which we have predicted infection risk, were defined using the reservoir and vector ranges generated by our earlier work and these ranges reflect the fact that species distributions are not fixed. Specifically, these ranges included introduced populations of the two macaque species, for example, pet *M*. *fascicularis* and *M*. *nemestrina* macaques are commonly found on Sulawesi where the environment is predicted to be suitable for the establishment of feral populations [26]. The predictions for infection risk that we present here therefore include locations on this island.

Human *P*. *knowlesi* infections have been identified beyond the ranges of both *M*. *nemestrina* and *M*. *fascicularis*. *Macaca leonina*, whose range extends farther north into Myanmar where these human cases were reported [58], has thus been implicated as a putative host species. We allowed predictions within the range of *M*. *leonina* but since this species is not found in MBS, it was not used as an explanatory covariate for model fitting. This may have impacted model predictions in the most northern parts of our study area where the environmental suitability for *M*. *nemestrina* and *M*. *fascicularis* is low, but high for *M*. *leonina*. Again, sampling in these areas, particularly northern Myanmar, would improve the predictions.

Furthermore, two distinct *P*. *knowlesi* parasite populations, linked to *M*. *nemestrina* and *M*. *fascicularis* respectively, have been identified in human patients from Malaysia [59]. It is reasonable to assume that only the strain associated with *M*. *fascicularis* is circulating and infecting humans in areas of continental Asia, where *M*. *nemestrina* is absent, and it may have a distinct relationship with environmental and socioeconomic variables compared to the mixture of parasite infections in patients from Malaysia. The presence of Leucosphyrus Complex vectors in Malaysia and Dirus Complex vectors in continental Asia [26] further adds to the possibility of different relationships between disease risk and the environment in these two regions.

Comparing our predicted *P*. *knowlesi* risk map (Fig 3A) with the map of current sampling efforts (Fig 2), and the map of malaria eliminating countries (Fig 3B), allows us to identify relative surveillance priorities for *P*. *knowlesi*. These include a number of regions in Thailand (Phisanulok, Phetchuban, Chaiyaphum, Prachan Buri, and southern Nakhon Ratchasima) and Vietnam (Lam Dong, Phu Yen, Gia Lai, and Kon Tum). We also propose that further surveillance in previously sampled areas of Thailand, Vietnam and Cambodia is required to fully understand the distribution of *P*. *knowlesi* in countries close to eliminating the human malarias.

Among the countries next expected to eliminate the human malarias, our results highlight a need for surveillance in un-sampled, high-risk areas in Myanmar, Laos, and Sumatra and Kalimantan in Indonesia. Initial studies have reported cases in Aceh on Sumatra [13], and South and Central Kalimantan [60, 61] but no published reports are available from the other parts of these regions. Further surveillance is also needed in previously sampled areas, including Palawan in the Philippines.

Importantly, our map predicts the environmental suitability for infection, not the prevalence of infection or incidence of cases in these places. The higher numbers of reported cases in Malaysia is not proof that the disease risk is higher here because most locations outside Malaysia simply have not been surveyed and *P*. *knowlesi* could be misdiagnosed as one of the human malarias. Studies that have investigated numbers of cases or infections have sampled a wide array of communities including malaria patients [4], patients diagnosed as *P*. *malariae* by microscopy [62], and whole communities [63], meaning the disease prevalence indicators generated are not directly comparable. Until more locations are surveyed using a consistent measurement (ideally infections in a cross section of the community) and diagnostics that distinguish all human malarias, we cannot draw any firm conclusions about relative disease prevalence [64]. Studies of other diseases have, however, found a relationship between the environmental suitability for infection and infection prevalence or case incidence [17, 65] and this is a potential use of future iterations of this map. It will be important to update the predictions presented here when new data become available, and systems are available to generate updated predictive maps [66]. Importantly the map presented here provides key information about the locations where new surveys for *P*. *knowlesi* infections would be most informative.

As the volume and quality of geographical data on *P*. *knowlesi* infections increases across Southeast Asia, these maps will iteratively improve. For now, the work presented here provides the best evidence-base currently available for prioritizing *P*. *knowlesi* surveillance to better understand its spatial distribution and its wider contribution to malaria cases.

## Supporting Information

S1 File Methods supplement. (DOCX)

S2 File Database of *Plasmodium knowlesi* infections recorded in humans, macaque reservoirs and vectors. (XLSX)

S1 Fig Covariates used in predicting the distribution of risk of *Plasmodium knowlesi* malaria. **A.** Displays human population density. **B-D.** Show the relative environmental suitability for vector (the Leucosphyrus Group) and reservoir species (*Macaca fascicularis* and *M*. *nemestrina*) of *P*. *knowlesi*, respectively. **E.** Shows an index of temperature suitability for *P*. *falciparum* transmission. **F** and **G.** Display values for tasselled cap wetness, which is measure of surface moisture (mean and standard deviation, respectively). **H.** Displays standard deviation values for tasselled cap brightness, which is a measure of moisture on bare surfaces. **I.** Gives the time required to travel from each geographic location to a large city via land or water-based transport networks. **J.** Displays elevation. For details of how each of these covariates layers was derived see S1 File.
(TIF)

S2 Fig Land cover covariates used in predicting the distribution of risk of *Plasmodium knowlesi* malaria. **A-I.** Displays proportional cover for 2012 of lands with croplands, croplands natural vegetation mosaics, open shrublands, permanent wetlands, grasslands, intact forest, disturbed forest, woody savannas and savannas, respectively. For details of how each of these covariates layers was derived see S1 File.
(TIF)

S3 Fig Fine-scale map of presumed limits of zoonotic *Plasmodium knowlesi* transmission. Areas of spatial co-occurrence of known or putative reservoir (at least one of *M*. *fascicularis*, *M*. *nemestrina* or *M*. *leonina*) and vector species (members of the Leucosphyrus Group) are indicated, as well as areas where either reservoir or vector species are absent.
(TIF)

S4 Fig Map of model uncertainty. Standard deviation values for each pixel were calculated across the model ensemble. Areas from lower to higher standard deviation values are shown.
(TIF)

S5 Fig Unmasked mean model output. Suitability for zoonotic *Plasmodium knowlesi* transmission from known reservoir and vector species from relative low to high suitability.
(TIF)

S6 Fig Predicted disease risk values at locations with confirmed/unconfirmed presence and absence from outside Malaysia, Brunei and Singapore. The black dots represent the predicted values of confirmed/unconfirmed *P*. *knowlesi* presence and absence points and violin plots showing the density of points at each predicted value are shown in grey. Reports of *P*. *knowlesi* that were not supported by results from a second independent group working in the same region were classified as unconfirmed.
(TIF)

S7 Fig Marginal effect plots for the most influential covariates. The black line represents the mean marginal effect and grey envelopes the associated 95% quantiles. The mean relative contribution is displayed in the top left corner of each plot.
(TIF)
