# Supplementary material for: Estimating Geographical Variation in the Risk of Zoonotic Plasmodium knowlesi Infection in Countries Eliminating Malaria
Source: PLoS Negl Trop Dis. 2016 Aug 5;10(8):e0004915. doi: 10.1371/journal.pntd.0004915 (PMC4975412; doi:10.1371/journal.pntd.0004915)
Supplement: S1 File — (DOCX) [file pntd.0004915.s001.docx]

**Methods Supplement**

**Assembly of the occurrence dataset**

We undertook a comprehensive literature search for studies that used diagnostics with the capacity to detect *Plasmodium knowlesi* in humans, non-human primates and mosquitoes, and then applied inclusion criteria to ensure the data quality met our minimum standards.

First, we conducted a Web of Science bibliography search using the terms “knowlesi” and “monkey malaria”. We also searched for “malaria” in combination with each country name from the region. This returned 1,722 articles. We then excluded all laboratory studies and examined the main text of each of the remaining articles and retained those reporting studies that had used either 1) *P. knowlesi*-specific molecular identification methods, for example the use of semi-nested PCR primers [1] or 2) a combination of microscopy and molecular techniques that distinguish *P. knowlesi* from *P. falciparum* and *P. malariae*. This led to a total of 72 articles. We contacted the authors of each of these studies to request any information we required which was not provided in the article, and to identify additional unpublished datasets.

In addition to this literature search, we contacted 12 researchers who use molecular techniques to survey malaria parasites in the region (typically projects looking for drug resistance genes) and determine whether they had detected *P. knowlesi* in any of these samples.

Whilst we did not limit the temporal range of our literature search, the first studies that met our inclusion criteria for identification of *P. knowlesi* were published in 2004 [2, 3] so our literature search included articles published from January 2004 to October 2015 when the search was completed. However some of the identified studies retrospectively analysed stored samples so the earliest identified infections in our database are from 1996. The studies which first described and investigated *P. knowlesi* in the 1950s and 1960s were not included because our aim was to map the contemporary distribution of this disease and there is evidence that current parasite populations are distinct from those isolated from these early cases [4].

The data obtained was disaggregated to each location and year sampled wherever possible. For each sample set collected at a unique location and time from either humans, macaques or mosquitoes, the following fields were recorded: date of collection, location of collection, number of samples tested with a knowlesi-specific diagnostic, the community sampled, whether the report came from a planned study of *P. knowlesi* or was an opportunistic report (*e.g.* case reports from travellers or patients with notable symptoms), the diagnostics used, the number of samples tested, and whether *P. knowlesi* was detected. Geographical coordinates were assigned to each location using a combination of information from the article and authors, and online gazetteers such as Google Maps and Geonames. If samples were obtained from a health facility, the location was defined as the administrative division most closely linked to the facility catchment area.

At the end of the initial data collation process the dataset was subjected to a final screen. Reports of *P. knowlesi* that were not supported by results from a second independent group working in the same region (samples less than 300 km apart) were marked as “unconfirmed”. These records were retained in the database, but not used in fitting or quantitatively evaluating the niche model. Additionally, reports linked to locations representing areas greater than 25,000 km^2^ are included in the database but were not included in the model training data.

**Explanatory covariates**

The environmental covariates included measures of the annual mean and standard deviation values for each pixel for tasseled cap brightness (TCB), tasseled cap wetness (TCW) and enhanced vegetation index (EVI). All these were derived from NASA’s moderate resolution imaging spectrometer (MODIS) satellite imagery [5, 6]. They were computed from the original 1 km × 1 km dataset and gap-filled using the Weiss *et al.* algorithm to model missing data caused by cloud cover [7]. Since TCB, TCW and EVI are all related to surface moisture (*i.e.* TCW is positively correlated with surface water, and EVI is related to vegetation density and thus positively correlated with rainfall in tropical and temperature regions), we calculated pairwise Spearman correlation coefficients to assess whether statistical dependence existed between two or more of these covariates. Statistical dependence can lead to unstable parameter estimates with inflated standard errors when covariate values have correlation coefficients of | *ρ* | ˃ 0.7 [8]. The pairwise Spearman coefficients were all greater than this threshold between: EVI mean and TCB mean; EVI standard deviation and TCB standard deviation; and TCW mean and TCB mean. We therefore removed TCB mean, EVI mean and EVI standard deviation from our covariate dataset.

We also included a data surface of temperature suitability for *P. falciparum* transmission. It has been shown that an index of temperature suitability for malaria transmission that incorporates the relationships between temperature and both sporogeny and adult mosquito longevity is a better predictor of disease risk than temperature alone [9]. For this reason a temperature suitability index for *P. falciparum* transmission [10] was included, in the absence of the biological data needed to develop a *P. knowlesi* transmission temperature suitability index.

Land cover covariates were derived from the International Geosphere-Biosphere land cover classification available within the MODIS MCD12Q1 dataset at a 500 m × 500 m spatial resolution [11]. The 500 m × 500 m categorical datasets were summarized to produce a value for proportional land cover of either open shrublands, woody savannas, savannas, grasslands, permanent wetlands, croplands or cropland mosaics within the larger 5 km × 5 km grid cells. Annual data surfaces were calculated using data for each year from 2001 to 2012.

Separate intact forest and disturbed forest data surfaces that track the decline in forest cover and any areas of regrowth from 2001 to 2012 were also constructed using annual data for the first five forest classes in the International Geosphere-Biosphere land cover dataset. The values range from 0.0 (no forest cover) to 1.0 (complete forest cover). The development of these layers is described in detail elsewhere [12]. Additional information on covariates derived from satellite imagery and their subsequent processing, is provided elsewhere [9].

Other covariates include human population density, urban accessibility and elevation. Fine scale human population density data for 2010 was obtained from WorldPop [13] and Gridded Population of the World [14]. These two data sets were mosaiced together, with WorldPop data used in preference where they overlapped. Then the sum of the 1 km × 1 km pixels in each 5 km × 5 km pixel was calculated to produce the final surface. The urban accessibility index was calculated using data from the European Commission Joint Research Centre Global Environment Monitoring Unit giving the time required to travel from a given pixel to an urban settlement of 50,000 people or more via land or water-based transportation networks [15]. We took the reciprocal of this quantity and re-scaled it between 0 and 1 such that high values of urban accessibility index indicated less travel time to a city. More detailed information on the construction of the urban accessibility layer is provided elsewhere [16]. The elevation covariate was derived from the Shuttle Radar Topography Mission (SRTM) dataset [17], with the original 90 m spatial resolution data summarized to produce a 5 km × 5 km surface [9].

Finally, we included two fine scale species distributions for the known macaque reservoirs of *P. knowlesi*, *Macaca fascicularis* and *M. nemestrina*, and one for members of the Leucosphyrus Group, which have been implicated as *P. knowlesi* vectors. Separate distribution maps were produced for each year from 2001 to 2012. The development of these species distribution surfaces is described in detail elsewhere [12].

Since the original data for our covariates came from a variety of sources, all covariate grids were standardized to ensure identical spatial resolution, extent and boundaries.

**Model fitting**

To increase the robustness of model predictions and quantify model uncertainty, 500 sub-models were fitted with each trained to a separate bootstrap dataset randomly sampled with replacement from the complete presence/background dataset. Each bootstrap had the same number of data points as the complete dataset and contained a minimum of ten presence and ten background points. To incorporate uncertainty in the location of infection for polygon occurrence records, each bootstrap drew a randomly selected 5 km × 5 km pixel within each polygon. This results in a Monte Carlo simulation to integrate across uncertainty in the spatial location of the samples, assuming that it is equally likely that the samples arose from each pixel. Likelihood weights were applied to the background points such that the sum of weights of the background data plus the number of absence records equaled the number of presence records for each bootstrap dataset. This has been shown to improve model predictive accuracy where unbiased presence-absence data are unavailable [18]. The algorithm hyper-parameters were set to the following values: cross-validation folds=10, tree complexity=4, learning rate=0.005, bag fraction=0.75, step size=10.

Since the occurrence dataset includes data from humans, macaques and mosquitoes, a joint model was fitted for human, macaque and mosquito hosts that enabled all available infection data to be leveraged, whilst not constraining the model to assume that the distribution of infection risk would be identical for all three host organisms. As BRTs can fit high-dimensional interactions, the joint model is then able to fit different environmental responses for each host organism, or if there is no difference in the signal, to fit the same response for all of them. To make predictions, an additional covariate data surface was generated for each host species, where the value of every pixel equalled 1, 2 or 3, to represent mosquito, macaque and human hosts, respectively. Then three separate predictions were made, each using the complete set of environmental and socio-economic data surfaces, as well as the relevant host data surface (refer to the R code for further details (link to be insert on acceptance)).

The model did not detect appreciable differences in the environmental predictors of infection risk in humans, macaques or mosquitoes. The model of human infection risk was therefore used for final model outputs.

**Model evaluation**

The model’s predictive performance was evaluated using the area under the receiver operator curve (AUC) statistic, *i.e.* the area under a plot of the true positive rate versus false positive rate for varying prediction thresholds, reflecting the ability to discriminate between presence and background records [19]. For each sub-model, the mean AUC under 10-fold cross validation was calculated using a pairwise distance sampling procedure to remove potential spatial sorting bias in the cross validation datasets. This AUC was then averaged across all 500 sub-models in the ensemble to give an overall estimate of predictive performance, as well as uncertainty in this estimate.

To assess the model’s predictive performance outside its training region, a separate AUC value was calculated for each sub-model using a validation dataset including presence and absence records from locations outside Malaysia, Brunei and Singapore. Only presence and absence records marked as “confirmed” (see **Assembly of the occurrence database**) were included in the validation dataset. This AUC was also calculated for each sub-model and then averaged across all 500 sub-models in the ensemble.

**References**

1. Imwong M, Tanomsing N, Pukrittayakamee S, Day N, White N, Snounou G. Spurious amplification of a *Plasmodium vivax* smallsubunit RNA gene by use of primers currently used to detect *P. knowlesi*. J Clin Microbiol. 2009;47:4173-5.

2. Singh B, Sung LK, Matusop A, Radhakrishnan A, Shamsul SSG, Cox-Singh J, et al. A large focus of naturally acquired *Plasmodium knowlesi* infections in human beings. The Lancet. 2004;363(9414):1017-24.

3. Jongwutiwes S, Putaporntip C, Iwasaki T, Sata T, Kanbara H. Naturally acquired *Plasmodium knowlesi* malaria in human, Thailand. Emerg Infect Dis. 2004;10(12):2211.

4. Assefa S, Lim C, Preston MD, Duffy CW, Nair MB, Adroub SA, et al. Population genomic structure and adaptation in the zoonotic malaria parasite *Plasmodium knowlesi*. Proc Natl Acad Sci USA. 2015;112(42):13027-32.

5. Lobser S, Cohen W. MODIS tasselled cap: land cover characteristics expressed through transformed MODIS data. Int J Remote Sens. 2007; 28:5079-101.

6. Huete A, Justice C, Van Leeuwen W. MODIS vegetation index (MOD13). Algorithm theoretical basis document. 1999.

7. Weiss DJ, Atkinson PM, Bhatt S, Mappin B, Hay SI, Gething PW. An effective approach for gap-filling continental scale remotely sensed time-series. ISPRS J Photogram Remote Sens. 2014;98:106-18.

8. Dormann CF, Elith J, Bacher S, Buchmann C, Carl G, Carré G, et al. Collinearity: a review of methods to deal with it and a simulation study evaluating their performance. Ecography. 2013;36(1):27-46.

9. Weiss DJ, Mappin B, Dalrymple U, Bhatt S, Cameron E, Hay SI, et al. Re-examining environmental correlates of *Plasmodium falciparum* malaria endemicity: a data-intensive variable selection approach. Malar J. 2015;14(1):574-.

10. Gething PW, Van Boeckel TP, Smith DL, Guerra CA, Patil AP, Snow RW, et al. Modelling the global constraints of temperature on transmission of *Plasmodium falciparum* and *P. vivax*. Parasit Vectors. 2011;4(1):1-11.

11. Friedl MA, Sulla-Menashe D, Tan B, Schneider A, Ramankutty N, Sibley A, et al. MODIS Collection 5 global land cover: Algorithm refinements and characterization of new datasets. Remote Sens Environ. 2010;114(1):168-82.

12. Moyes CL, Shearer FM, Huang Z, Wiebe A, Gibson HS, Nijman V, et al. Predicting the geographical distributions of the macaque hosts and mosquito vectors of *Plasmodium knowlesi* malaria in forested and non-forested areas. Parasit Vectors. 2016;9(1):242.

13. WorldPop. Gridded Population Distributions. 2015 [Accessed: July 2015]. Available from: <http://www.worldpop.org.uk/data/summary/?contselect=Asia&countselect=Whole+Continent&typeselect=Population+2010>.

14. Center for International Earth Science Information Network - CIESIN - Columbia University, United Nations Food + Agriculture Programme - FAO, Centro Internacional de Agricultura Tropical - CIAT. Gridded Population of the World, Version 4 (GPWv4): Population Count Grid. Palisades, NY: NASA Socioeconomic Data and Applications Center (SEDAC); 2005.

15. Joint Research Center Global Environmental Modelling Unit. Travel time to major cities: A global map of accessibility. Available from: <http://bioval.jrc.ec.europa.eu/products/gam/sources.htm>.

16. Bhatt S, Gething PW, Brady OJ, Messina JP, Farlow AW, Moyes CL, et al. The global distribution and burden of dengue. Nature. 2013;496(7446):504-7.

17. Farr TG, Rosen PA, Caro E, Crippen R, Duren R, Hensley S. The Shuttle Radar Topography Mission. Rev Geophys. 2007;45:RG2004.

18. Barbet-Massin M, Jiguet F, Albert CH, Thuiller W. Selecting pseudo-absences for species distribution models: how, where and how many? Methods Ecol Evol. 2012;3(2):327-38.

19. Fleiss J, Levin B, Paik M. Statistical methods for rates and proportions. Hoboken, New Jersey: John Wiley & Sons; 2003.
