# Supplementary figures and images for: Estimating Geographical Variation in the Risk of Zoonotic Plasmodium knowlesi Infection in Countries Eliminating Malaria
Source: PLoS Negl Trop Dis. 2016 Aug 5;10(8):e0004915. doi: 10.1371/journal.pntd.0004915 (PMC4975412; doi:10.1371/journal.pntd.0004915)

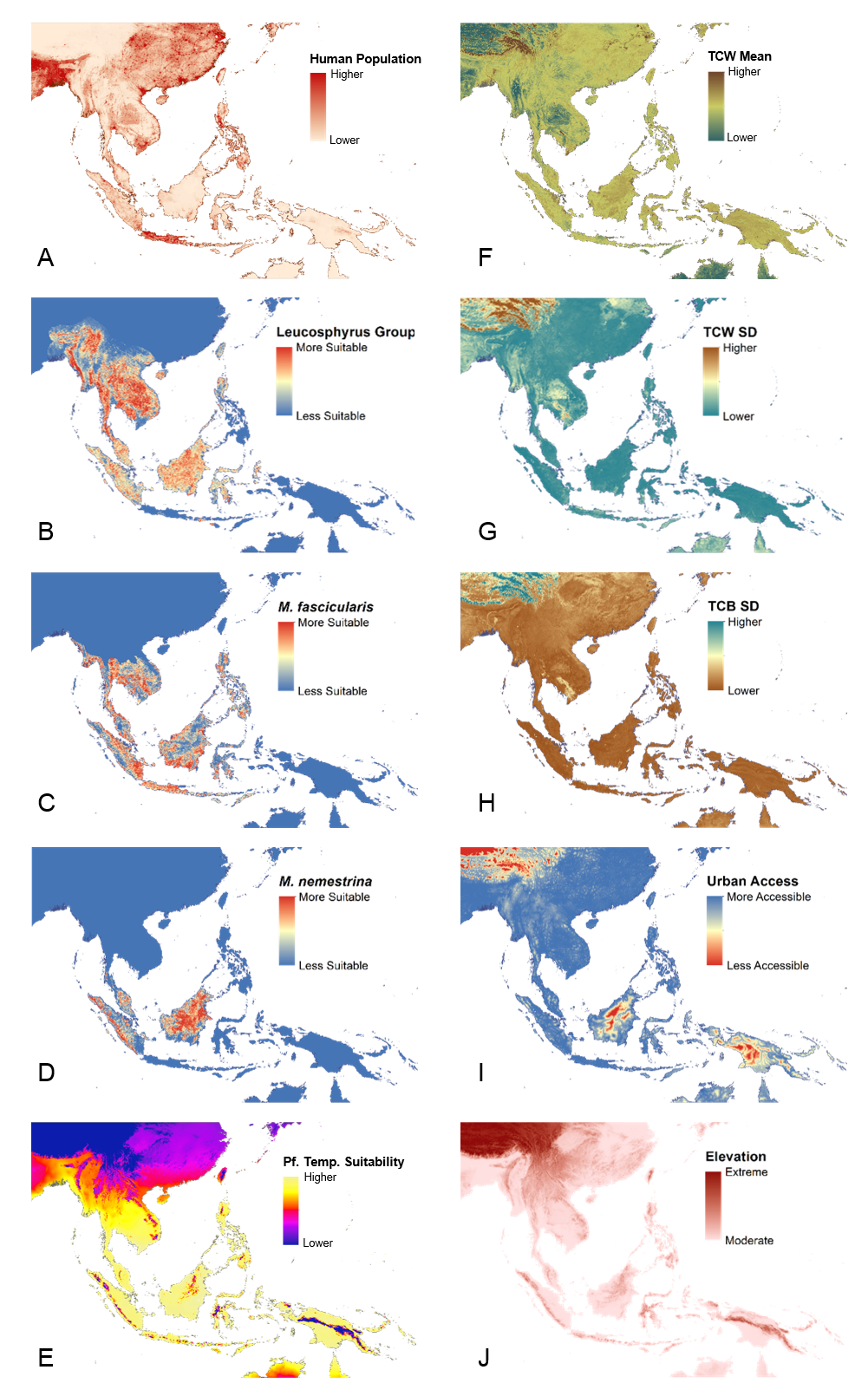

Supplement: S1 Fig — A. Displays human population density. B-D. Show the relative environmental suitability for vector (the Leucosphyrus Group) and reservoir species (Macaca fascicularis and M. nemestrina) of P. knowlesi, respectively. E. Shows an index of temperature suitability for P. falciparum transmission. F and G. Display values for tasselled cap wetness, which is measure of surface moisture (mean and standard deviation, respectively). H. Displays standard deviation values for tasselled cap brightness, which is a measure of moisture on bare surfaces. I. Gives the time required to travel from each geographic location to a large city via land or water-based transport networks. J. Displays elevation. For details of how each of these covariates layers was derived see S1 File. (TIF) [file pntd.0004915.s003.tif]

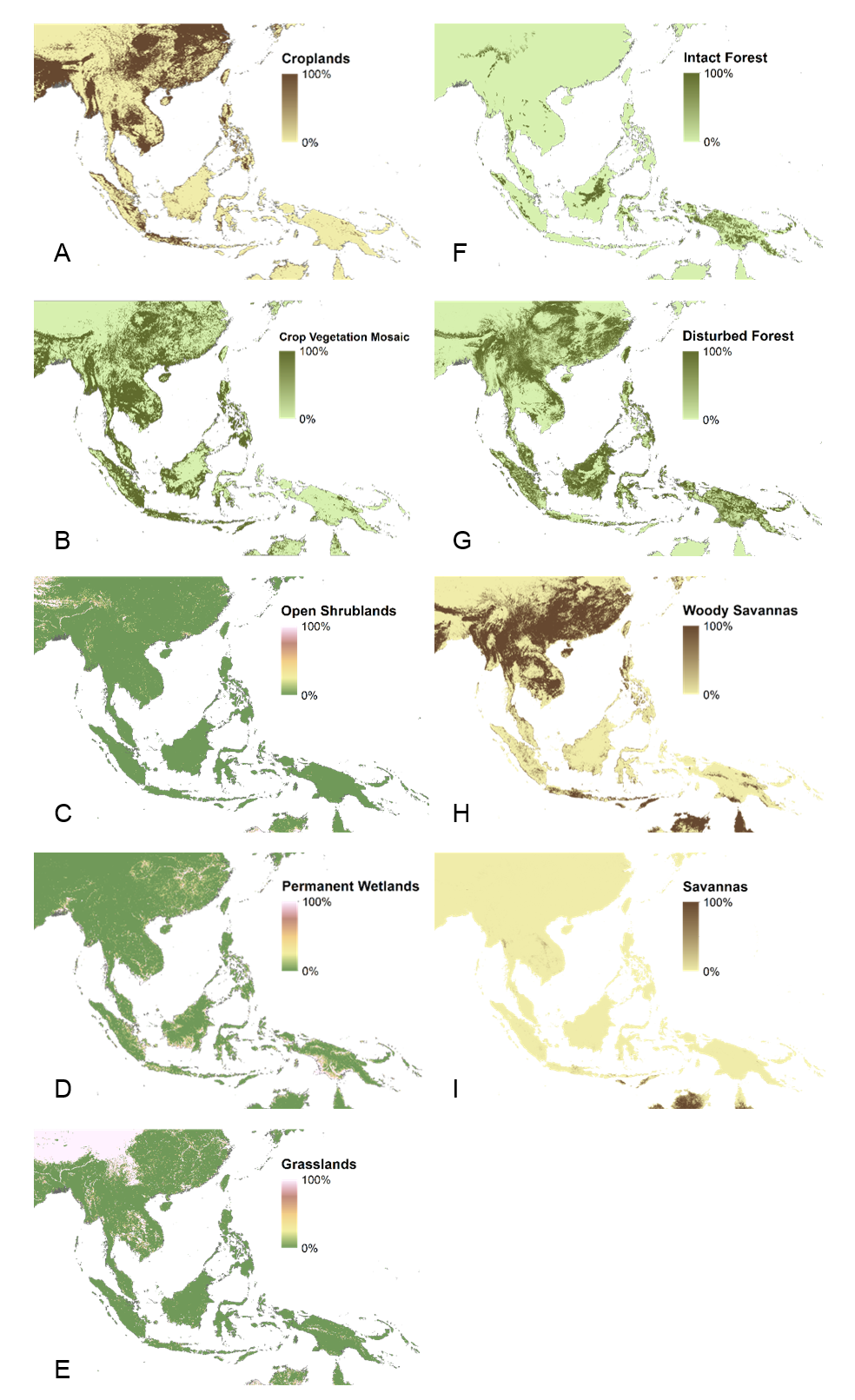

Supplement: S2 Fig — A-I. Displays proportional cover for 2012 of lands with croplands, croplands natural vegetation mosaics, open shrublands, permanent wetlands, grasslands, intact forest, disturbed forest, woody savannas and savannas, respectively. For details of how each of these covariates layers was derived see S1 File. (TIF) [file pntd.0004915.s004.tif]

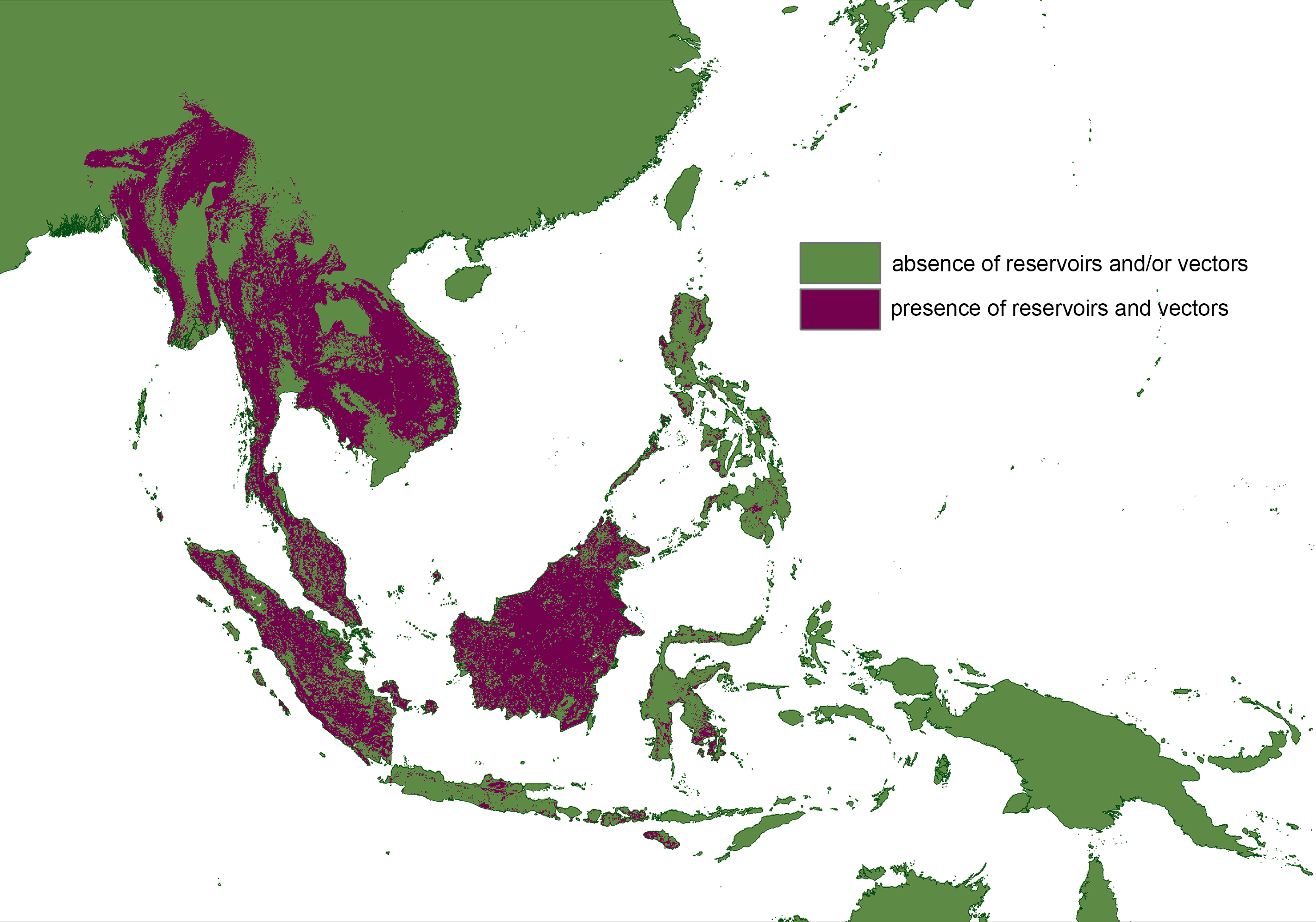

Supplement: S3 Fig — Areas of spatial co-occurrence of known or putative reservoir (at least one of M. fascicularis, M. nemestrina or M. leonina) and vector species (members of the Leucosphyrus Group) are indicated, as well as areas where either reservoir or vector species are absent. (TIF) [file pntd.0004915.s005.tif]

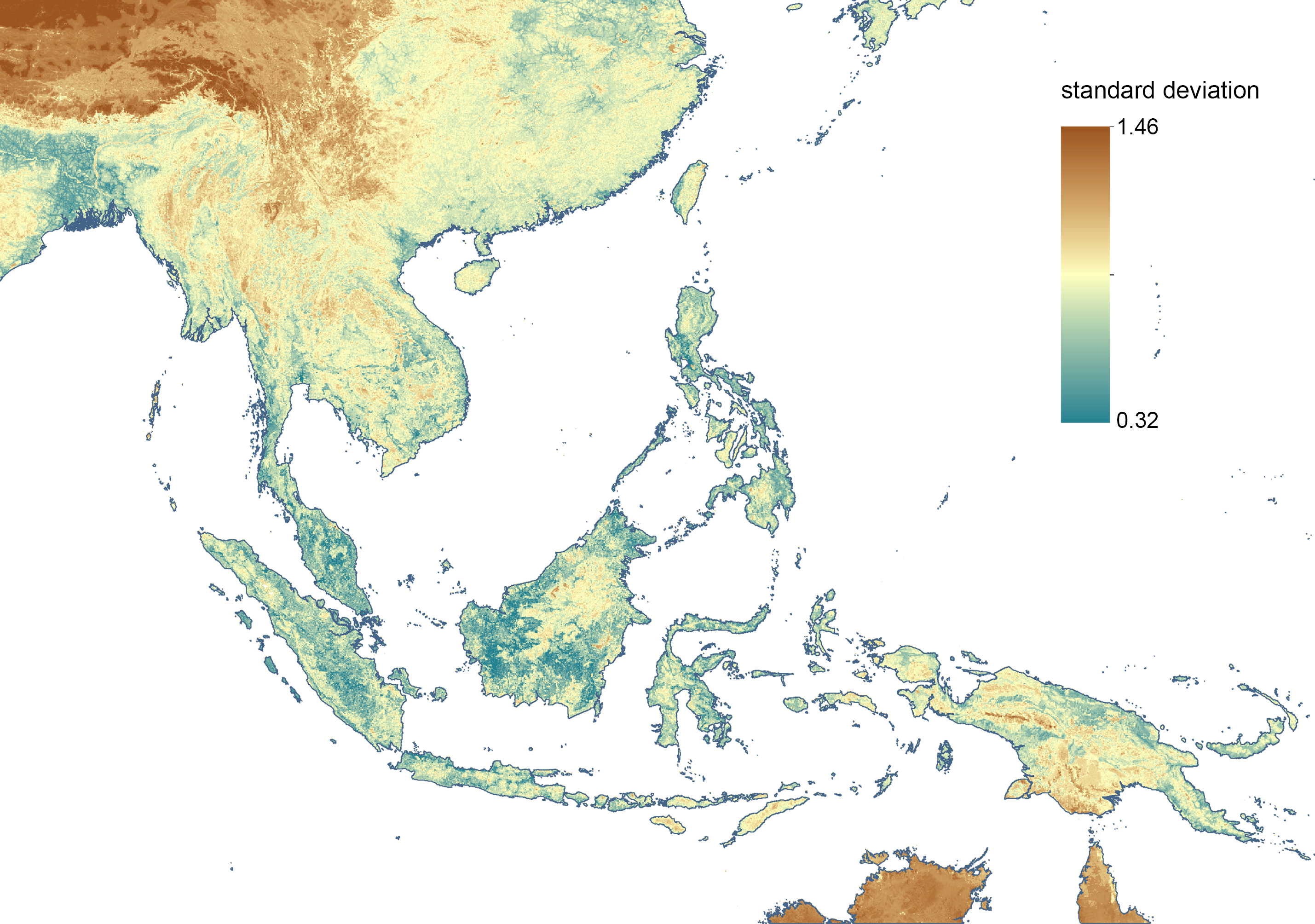

Supplement: S4 Fig — Standard deviation values for each pixel were calculated across the model ensemble. Areas from lower to higher standard deviation values are shown. (TIF) [file pntd.0004915.s006.tif]

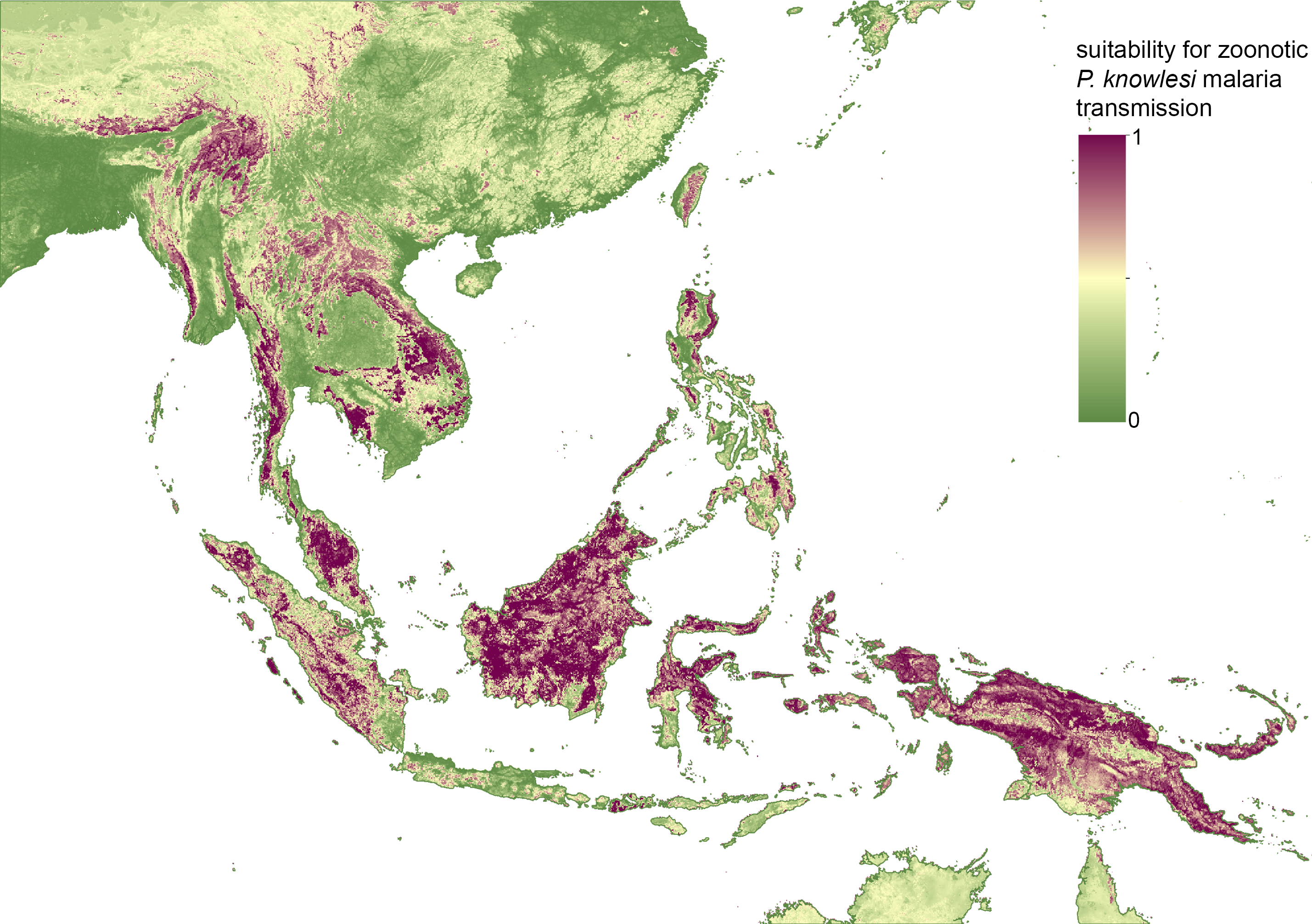

Supplement: S5 Fig — Suitability for zoonotic Plasmodium knowlesi transmission from known reservoir and vector species from relative low to high suitability. (TIF) [file pntd.0004915.s007.tif]

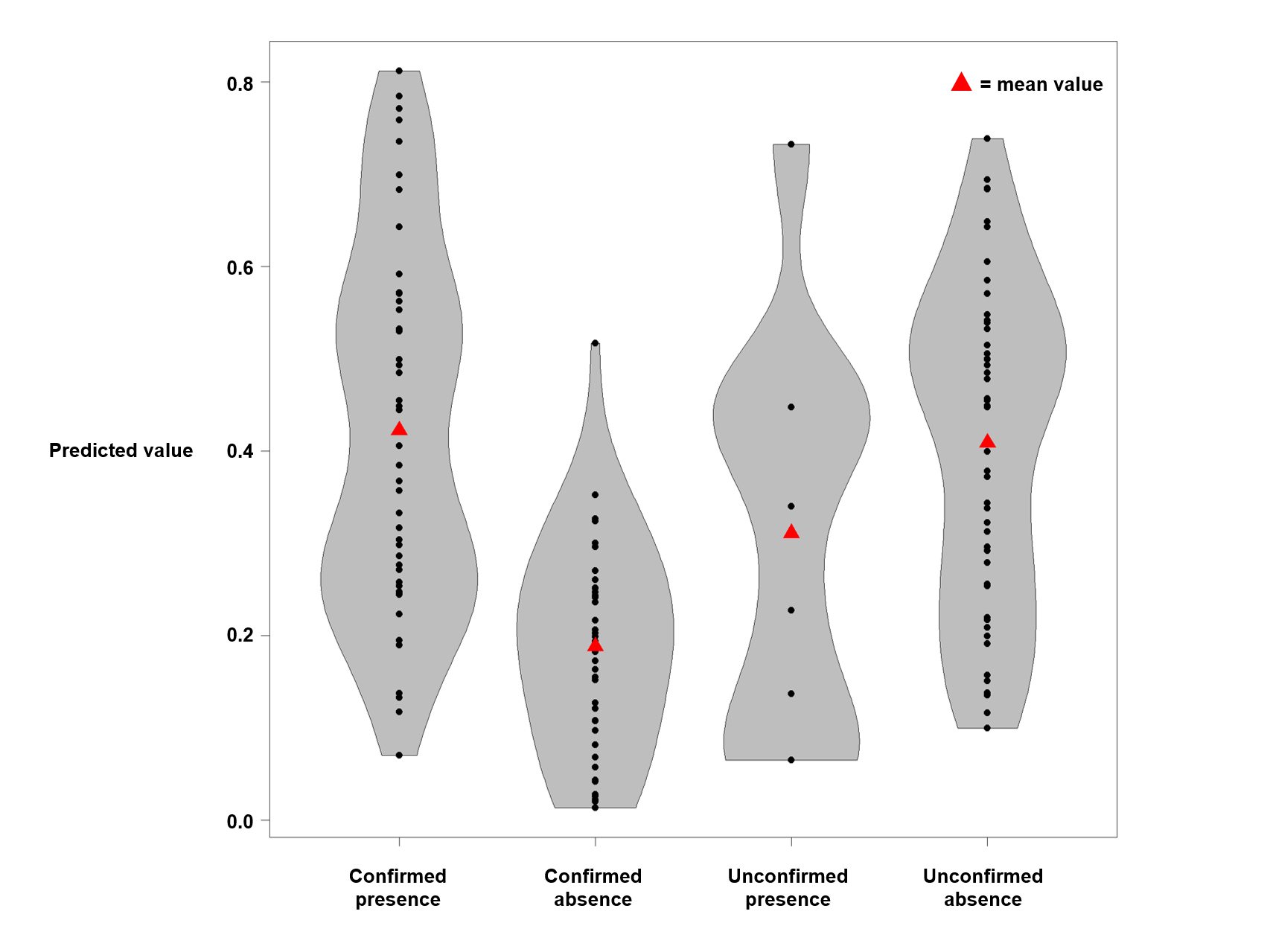

Supplement: S6 Fig — The black dots represent the predicted values of confirmed/unconfirmed P. knowlesi presence and absence points and violin plots showing the density of points at each predicted value are shown in grey. Reports of P. knowlesi that were not supported by results from a second independent group working in the same region were classified as unconfirmed. (TIF) [file pntd.0004915.s008.tif]

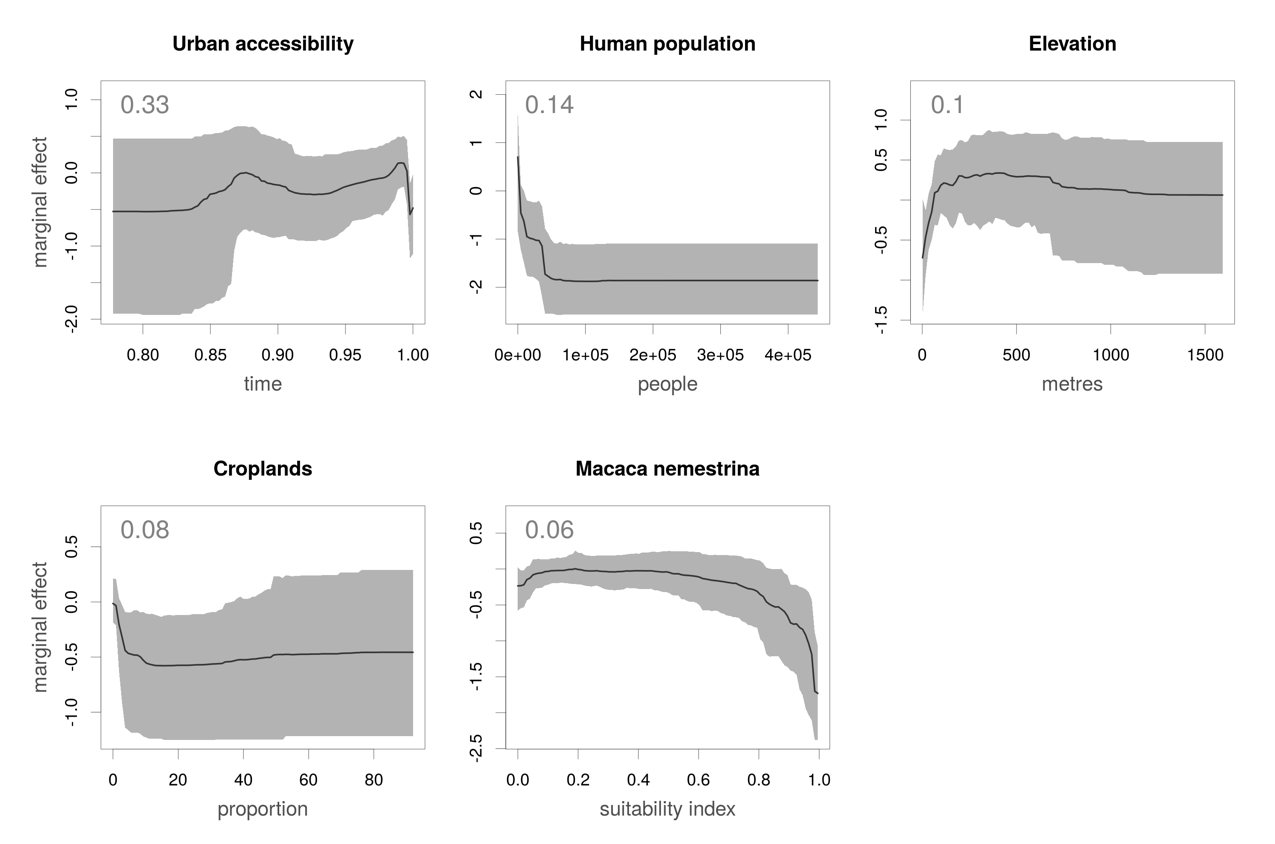

Supplement: S7 Fig — The black line represents the mean marginal effect and grey envelopes the associated 95% quantiles. The mean relative contribution is displayed in the top left corner of each plot. (TIF) [file pntd.0004915.s009.tif]
